# Supplementary material for: Elevational Variation in and Environmental Determinants of Fungal Diversity in Forest Ecosystems of Korean Peninsula
Source: J Fungi (Basel). 2024 Aug 7;10(8):556. doi: 10.3390/jof10080556 (PMC11355257; doi:10.3390/jof10080556)
Supplement: Supplementary file 1 [file jof-10-00556-s001.zip › jof-supplementary-figure-and-table.pdf]

Supplementary Material

Elevational Variation and Environmental Determinants of Fungal Diversity in Forest Ecosystems of Korean Peninsula

Lei Chen [1], Zhi Yu [2], Mengchen Zhao [1], Dorsaf Kerfahi [3], Nan Li [4], Lingling Shi [5], Xiwu Qi [6], Chang-Bae Lee [7,8], Ke Dong [9,*], Hae-In Lee [7,*] and Sang-Seob Lee [1,*]

[1] Department of Integrative Biotechnology, Sungkyunkwan University, Suwon, 16419, South Korea

[2] Department of Environmental Health Sciences, Graduate School of Public Health, Seoul National University, Seoul, 08826, South Korea

[3] Department of Biological Sciences, School of Natural Sciences, Keimyung University, Daegu, 42601, South Korea

[4] Key Laboratory of Climate, Resources and Environment in Continental Shelf Sea and Deep Sea of Department of Education of Guangdong Province, Department of Oceanography, Key Laboratory for Coastal Ocean Variation and Disaster Prediction, College of Ocean and Meteorology, Guangdong Ocean University, Zhanjiang, China

[5] Department of Geosciences, Geo-Biosphere Interactions, Faculty of Sciences, University of Tuebingen, Tuebingen, Germany

[6] Jiangsu Key Laboratory for the Research and Utilization of Plant Resources, Institute of Botany, Jiangsu Province and Chinese Academy of Sciences, Nanjing 210014, China; qixiwu@jib.ac.cn

[7] Biodiversity and Ecosystem Functioning Major, Department of Climate Technology Convergence, Forest Carbon Graduate School, Kookmin University, Seoul, Republic of Korea

[8] Department of Forestry, Environment and Systems, College of Science and Technology, Kookmin Uni-versity, Seoul, Republic of Korea

[9] Life Science Major, Kyonggi University, Suwon, 16227, South Korea

* Correspondence: dongke@kyonggi.ac.kr (K.D.); haein@kookmin.ac.kr (H.L.); syhwh@skku.edu (S.L.)

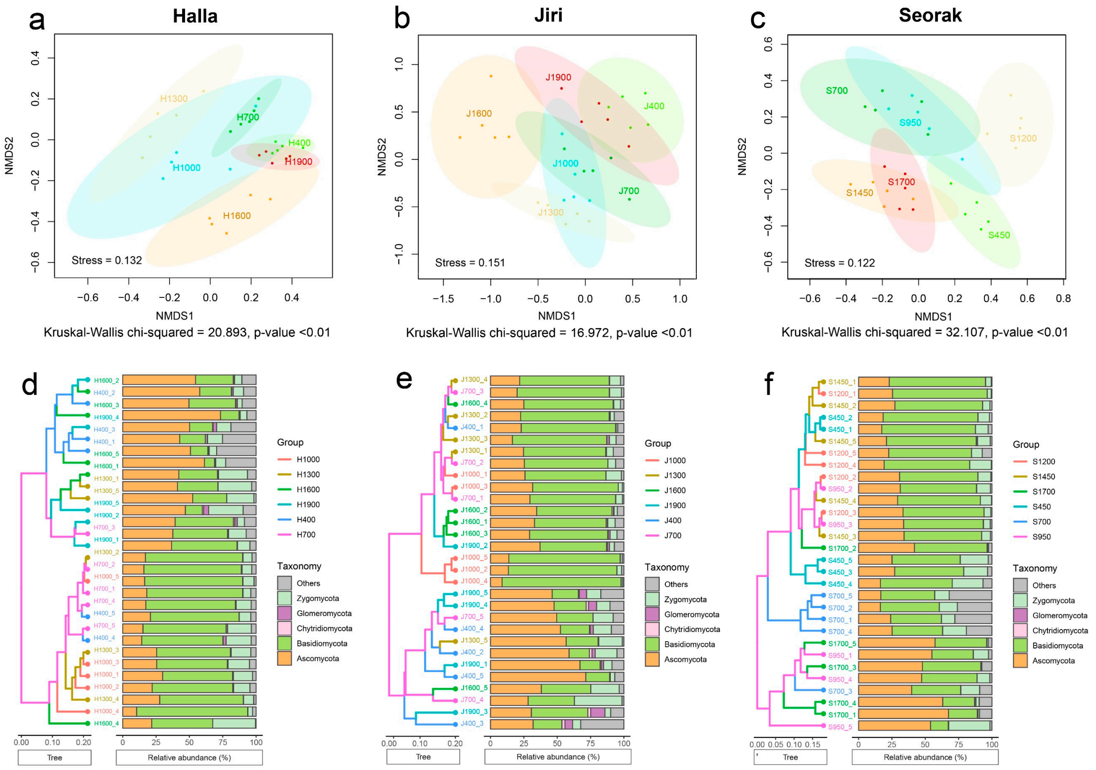

Figure S1. Composition analysis of fungal communities across different elevations on Mt. Halla, Mt. Jiri, and Mt. Seorak. a-c: Non-metric multidimensional scaling (nMDS) plots based on Bray-Curtis dissimilarity showing the clustering of fungal communities at different elevations. Each point represents a sample, and different colors indicate different elevation bands. A Kruskal-Wallis test was used to compare differences between elevations, with significant differences indicated at P<0.01. d-f: Hierarchical clustering dendrograms and corresponding bar plots showing the relative abundance of major fungal phyla at different elevations. The nMDS analysis results demonstrated the consistency of samples within each elevation band. The clustering analysis indicated that samples from the same elevation band within the same mountain tend to cluster together more closely than samples from different elevation bands.

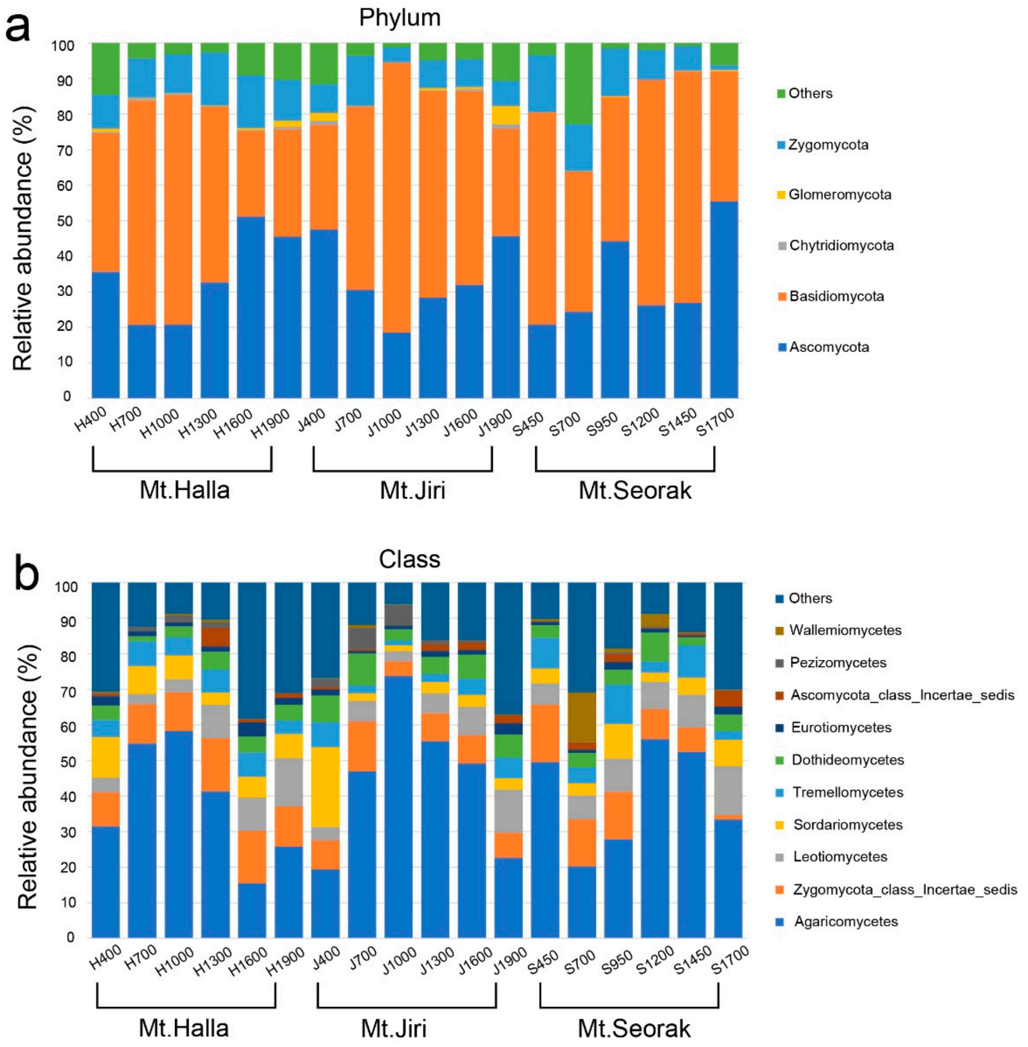

Figure S2. The relative abundance of fungal phyla and classes varied with elevation.

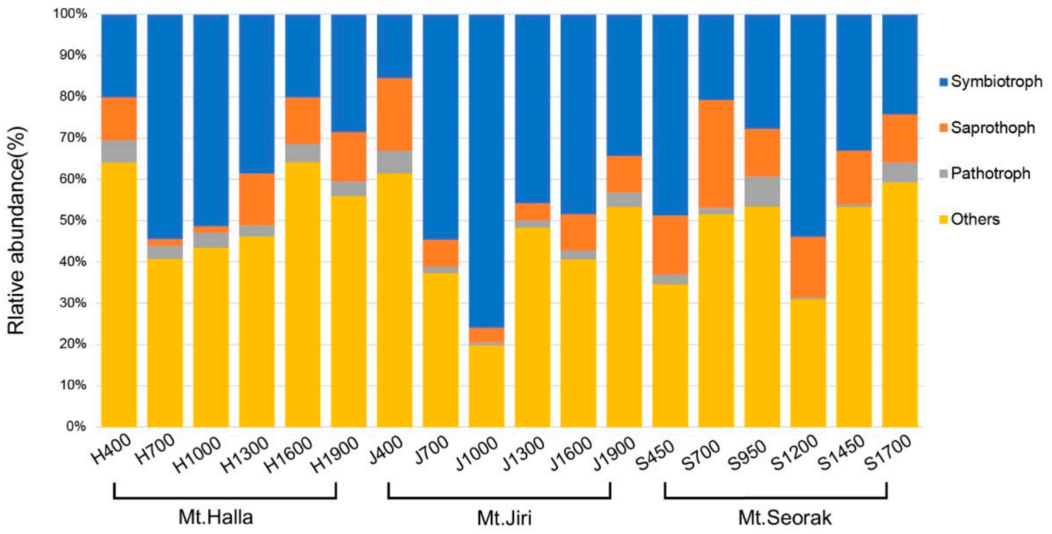

Figure S3. Changes in the relative abundance of fungal functional guilds along the elevation gradient across the three mountains.

Table S4. Compares the fitting performance of specific fungal phyla/classes across different regression models (linear regression, quadratic model, and cubic model). Model indicators include R^2, P values (*, $p < 0.05$; **, $p < 0.01$; ***, $p < 0.001$), and AIC. From top to bottom, the entries correspond to Mt. Halla, Mt. Jiri, Mt. Seorak.

Mountain	alpha diversity	Linear regression			Quadratic regression			Cubic regression		
		R^2	*p*	AIC	R^2	*p*	AIC	R^2	*p*	AIC
Halla	*Ascomycota*	0.0739	>0.05	88	0.1043	>0.05	89	0.1044	>0.05	91
	Basidiomycota	0.0011	>0.05	90	0.0397	>0.05	91	0.0756	>0.05	92
	Zygomycota	0.0104	>0.05	90	0.0208	>0.05	91	0.0814	>0.05	92
	Agaricomycetes	0.0182	>0.05	90	0.1131	>0.05	89	0.1684	>0.05	89
	Dothideomycetes	0.0165	>0.05	90	0.0252	>0.05	91	0.0252	>0.05	91
	Leotiomycetes	0.0945	>0.05	87	0.1752	>0.05	86	0.1820	>0.05	88
	Tremellomycetes	0.0479	>0.05	89	0.1171	>0.05	88	0.1173	>0.05	90
	Zygomycota_clas	0.0043	>0.05	90	0.0089	>0.05	92	0.1925	>0.05	88
	All	0.0772	>0.05	88	0.1057	>0.05	89	0.1940	>0.05	88
Jiri	*Ascomycota*	0.0785	>0.05	88	0.2748	*	82	0.5251	*	72
	Basidiomycota	0.0132	>0.05	90	0.1502	>0.05	87	0.1547	>0.05	89
	Zygomycota	0.0464	>0.05	89	0.0745	>0.05	90	0.0770	>0.05	92
	Agaricomycetes	0.0020	>0.05	90	0.0761	>0.05	90	0.0798	>0.05	92
	Dothideomycetes	0.0185	>0.05	90	0.2749	**	82	0.3809	**	80
	Leotiomycetes	0.1204	>0.05	86	0.1307	>0.05	88	0.1846	>0.05	88
	Tremellomycetes	0.2807	**	80	0.3770	*	78	0.3875	**	79
	Zygomycota_class_Incertae_sedis	0.1032	>0.05	87	0.1074	>0.05	89	0.1584	>0.05	89
	All	0.0884	>0.05	87	0.4556	***	74	0.4905	*	74
Seorak	*Ascomycota*	0.1423	*	86	0.1561	>0.05	87	0.1685	*	89
	Basidiomycota	0.0460	>0.05	89	0.1322	>0.05	88	0.1425	>0.05	90
	Zygomycota	0.1112	>0.05	87	0.1113	>0.05	89	0.2252	>0.05	86
	Agaricomycetes	0.0718	>0.05	88	0.1608	>0.05	87	0.1608	>0.05	89
	Dothideomycetes	0.0246	>0.05	89	0.0277	>0.05	91	0.0519	>0.05	93
	Leotiomycetes	0.0335	>0.05	89	0.0347	>0.05	91	0.0351	>0.05	93
	Tremellomycetes	0.1181	>0.05	86	0.1809	>0.05	86	0.1825	>0.05	88
	Zygomycota_class_Incertae_sedis	0.1324	*	86	0.1412	>0.05	88	0.3353	*	82
	All	0.1466	*	85	0.1515	>0.05	87	0.2758	*	84

Table S5. Compares the fitting performance of functional guilds across different regression models (linear regression, quadratic model, and cubic model). Model indicators include R^2, P values (*, $p < 0.05$; **, $p < 0.01$; ***, $p < 0.001$), and AIC. From top to bottom, the entries correspond to Mt. Halla, Mt. Jiri, Mt. Seorak.

Mountain	alpha diversity	Linear regression			Quadratic regression			Cubic regression		
		R^2	*p*	AIC	R^2	*p*	AIC	R^2	*p*	AIC
Halla	Pathotroph	0.0229	>0.05	89	0.0234	>0.05	91	0.0689	>0.05	92
	Saprotroph	0.0486	>0.05	89	0.0550	>0.05	90	0.3353	>0.05	82
	Symbiotroph	0.0101	>0.05	90	0.0945	>0.05	89	0.2090	>0.05	87
	All	0.0772	>0.05	88	0.1057	>0.05	89	0.1940	>0.05	88
Jiri	Pathotroph	0.0001	>0.05	90	0.2611	**	83	0.2616	>0.05	85
	Saprotroph	0.0273	>0.05	89	0.1681	>0.05	87	0.1785	>0.05	88
	Symbiotroph	0.0507	>0.05	89	0.1692	>0.05	87	0.1713	>0.05	88
	All	0.0884	>0.05	87	0.4556	***	74	0.4905	*	74
Seorak	Pathotroph	0.0788	>0.05	88	0.0790	>0.05	90	0.2736	>0.05	85
	Saprotroph	0.0848	>0.05	87	0.1655	>0.05	87	0.2755	>0.05	84
	Symbiotroph	0.0420	>0.05	89	0.1507	>0.05	87	0.2230	>0.05	87
	All	0.1466	*	85	0.1515	>0.05	87	0.2758	*	84
